# Application of the Acoustic Emission Method and Artificial Neural Networks to Assess the Damaging Effect of High Temperature on the Structure of Fibre-Cement Boards

**DOI:** 10.3390/ma15186460

**Published:** 2022-09-17

**Authors:** Tomasz Gorzelańczyk, Krzysztof Schabowicz, Mateusz Szymków

**Affiliations:** Faculty of Civil Engineering, Wrocław University of Science and Technology, Wybrzeże Wyspiańskiego 27, 50-370 Wrocław, Poland

**Keywords:** artificial neural networks, non-destructive testing, fibre-cement boards, acoustic emission, SEM

## Abstract

This article shows the results of research into the damaging effect of high temperature on the structure of fibre-cement boards. Samples of fibre-cement boards were exposed to high temperatures over various lengths of time and then they were investigated under the three-point bending and acoustic emission methods. In this way, the critical temperature and the duration of its influence on the structure of a fibre-cement board were determined. An artificial neural network was used to analyse the results obtained using the acoustic emission method. The investigations showed a marked fall in the number of registered AE events for the tested series of boards exposed to high temperature in comparison with the reference boards. Moreover, in the boards exposed to high temperature, a marked increase in the energy of AE events occurs during the bending test, whereby the registered events, by and large, come down to a single pulse induced by a brittle fracture. It is also demonstrated that the determination of the damaging effect of high temperature on the structure of fibre-cement boards solely on the basis of bending strength (*MOR*) is inadequate.

## 1. Introduction

Fibre-cement boards (FCB) have been used in buildings from the beginning of the twentieth century. This is a building product. The method of manufacturing this composite material was devised and patented by the Czech engineer Ludwik Hatschek, but the first such boards were with asbestos fibres. Asbestos fibres had been recognized to be carcinogenic, and they were replaced with synthetic or cellulose fibres [1]. The currently produced fibre-cement boards consist of synthetic fibres, cellulose fibres, cement and various innovative additives and admixtures, whereby they have become a completely different construction product. FCB have also other components and fillers: limestone flour, mica, pearlite, kaolin, microsphere, and also recycled materials [2,3], whereby FCB continues to be a very innovative material. It is also important for sustainable development and carbon footprint reduction [4]. FCB are used in construction mainly as rainscreen exterior wall cladding [5]. During use, FCBs are exposed to various environmental factors, chemical damage from acid rain and physical damage from ultraviolet radiation. Moreover, fibre-cement boards are exposed to operational and exceptional factors. These include, first of all, the high temperature produced by, e.g., a fire. Therefore, the determination of the degree of damage caused by high temperature is a major problem from both the scientific and practical points of view. A lot of the research to date on fibre-cement boards has dealt with the determination of their standard physico-mechanical properties, the effects of operational factors (such as wetting-drying cycles and freeze-thaw cycles), the effect of heating and sprinkling, the effect of high temperatures and the effect of using various types of fibres and production processes, solely on the basis of bending strength (the modulus of rupture (*MOR*)) [6]. In the literature, one can find few non-destructive investigations of fibre-cement boards and they describe only the imperfections arising during production [7,8,9,10]. The impact of high temperature is certainly one of the major damaging factors peculiar to many building products, particularly composite ones which contain reinforcement in the form of various kinds of fibres, especially cellulose fibres, as the latter undergo pyrolysis at temperatures above 200 °C. This has been experimentally demonstrated in studies [11,12,13]. Thus, high temperature greatly affects the durability of the whole composite [6]. To prove this thesis, experiments during which samples of fibre-cement boards were exposed to high temperatures ranging from 170 °C to 250 °C over a period of 0.5–4 h were carried out. After the exposure to high temperature the samples were investigated by registering the acoustic emission (EA) during three-point bending and then analysing the results using artificial neural networks (ANNs) [14]. Research conducted by the authors has shown that assessments of the degree of damage to fibre-cement boards caused by high temperature solely on the basis of bending strength (*MOR*) are inadequate [11,12,15,16]. By applying the acoustic emission method, it became possible to determine the effect of high temperature on the basis of the acoustic phenomena which may occur in the fibre-cement board. The registered AE signals were used to determine model acoustic spectrum characteristics which accompany the cracking of the cement matrix and the rupture of the fibres during bending. Then, recognition of the model characteristics in the EA recordings was carried out using artificial neural networks.

## 2. Survey of Literature

A review of literature on the subject shows that most of the research to date on FCB has been devoted to examining the effect of operational factors [17,18,19] and that of high temperatures by testing the physico-mechanical properties, mainly MOR, of such boards. In [20,21], nanoindentation was employed to assess the changes taking place in the structure of FCB under the influence of selected operating conditions. So far, there have been few works dealing with the investigation of FCB by means of non-destructive methods and the acoustic emission method. For example, [6] presents the results of research on fibre-cement boards, including the influence of high temperature, but based only on MOR. Furthermore, Li et al. [22] studied the effect of high temperatures on extruded composites on the basis of their mechanical properties. To assess the impact of high temperature and fire on fibre-cement boards, non-destructive methods such as acoustic emission were used, not only on the basis of physico-mechanical parameters in [11,12,23]. In the literature, one can also find other non-destructive studies of FCB, which deal mainly with the detection of imperfections arising at the production stage. In reference [7,24] by the authors present the possibility of using Lamb waves in a non-contact ultrasonic scanner to detect delamination and cracks in fibre-cement boards already at the production stage. Reference [25] describes a method of detecting delamination in materials using a mobile ultrasonic probe. Ultrasonic equipment and an idea for detecting delamination in FCB are described in [8] by Dębowski et al. In [26,27,28], it was proposed to use the impact-echo method and the impulse response method to discover delamination in concrete elements. However, it is not recommended to test fibre-cement boards using the two methods as they are intended for testing elements thicker than 100 mm, while the currently available fibre-cement boards are usually 8 mm thick. Furthermore, in the case of the impulse response method, a hammer strike can damage the board, while in the case of the impact-echo method, disturbances arise as a result of multiple reflections of waves, which makes the interpretation of the obtained image difficult, as described in [26]. In the literature, there is not enough information about the application of other non-destructive methods to the testing of FCB. Preliminary research reported by Chady et al. [10,29] confirmed the usefulness of the terahertz (t-ray) imaging method for testing FCB. Terahertz signals are similar in character to ultrasonic signals, but their interpretation is more complex. Adamczak-Bugno et al. [16] and Schabowicz et al. [30] used microtomography to identify delamination and low density regions in fibre-cement boards. The test results indicate that this method accurately distinguishes differences in the microstructure of the boards. However, this method can be applied only to small boards. As mentioned above, so far there have been few investigations into fibre-cement boards conducted using acoustic emission. Ranachowski et al. [31,32] carried out pilot studies of fibre-cement boards manufactured using extrusion and exposed as part of this process to the temperature of 230 °C. They used acoustic emission (AE) to determine the contribution of cellulose fibres to the strength of the boards and attempted to distinguish between the AE events emitted by the fibres and the ones emitted by the cement matrix. In their [11,12,15] the authors proposed to use the acoustic emission method to study the impact of fire and high temperature on fibre-cement boards. It should be mentioned that the influence of high temperature on concrete and the dependences involved were extensively investigated using acoustic emission by, e.g., Ranachowski et al. [33,34]. Melichar et al. [35] used the acoustic emission method to study modified cement-bonded particleboard under static load stress. During measurements performed using the acoustic emission method, a large amount of data are acquired. The data should be analysed and interpreted in a proper way. For this purpose, it can be useful to combine the AE method with artificial intelligence, including artificial neural networks (ANNs). Artificial neural networks have been successfully used to analyse and recognize signals obtained during the failure of different materials [36]. The most important feature of ANN is the parallel processing of information, which makes it possible to process large amounts of data and significantly speed up calculations. ANN was used to detect and recognize the characteristics of the acoustic spectra accompanying the cracking of the fibers or the cement matrix from the AE records during three-point bending. ANNs were used in [37,38,39] to analyse the results obtained from non-destructive tests of concrete. In [40,41] the acoustic emission method was used to test steel and artificial neural networks were employed to analyse the test results. Rucka and Wilde [42,43], Zielińska and Rucka [44] and Wojtczak and Rucka [45] successfully used the ultrasonic method to investigate damage to masonry structures. Finally, it should be mentioned that ANNs were also successfully employed to analyse the results of investigations of fibre-cement boards subjected to fire and freeze-thaw cycles [11,24].

Considering the above, the acoustic emission method combined with ANNs would be proper for assessing the damaging effect of high temperature on the structure of fibre-cement boards.

## 3. Strength Tests

High-temperature impact tests were carried out for five series of FCB designated with letters from A to E. The basic specifications of the boards in all the series, determined in accordance with the standard requirements [46] are presented in Table 1.

**Figure 1:**
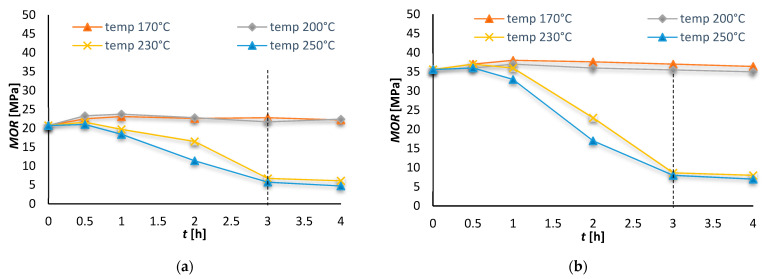
*MOR* of samples exposed to high temperature over time of 0.5–4 h: (**a**) series A, (**b**) series C.

The samples of fibre-cement boards were first exposed to high temperatures of 170–250 °C in a laboratory oven over times ranging from 0.5 to 4 h. The tests were first of all designed to indicate the “critical” temperature that has a significant effect on the bending strength (*MOR*) of the tested fibre-cement boards. The determined effect of selected temperatures on the *MOR* of series A and C samples is shown in Figure 1.

The diagrams in Figure 1 show that the influence of the temperature of 230 °C and higher on the bending strength (*MOR*) of the FCB is significant. On this basis, a critical temperature of 230 °C and its duration of 3 h were established. It is apparent that the above diagrams indicate only that as a result of the high temperature the MOR of the boards decreases, but they do not answer the question what changes occur in the structure of the tested boards. In order to find an answer to this question the authors carried out detailed investigations described further in this paper.

**Figure 2:**
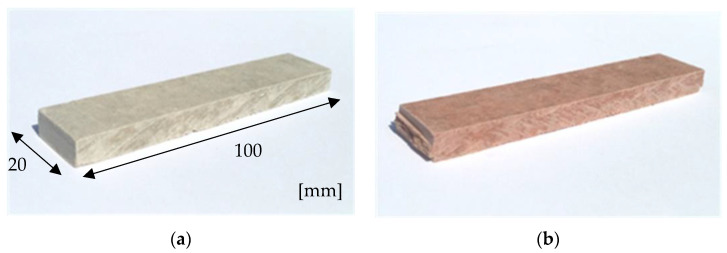
Tested fibre-cement board samples: (**a**) board A, (**b**) board C.

The tests aimed at determining the effect of the temperature of 230 °C lasting for 3 h on the structure of fibre-cement boards were carried out on samples designated A_T_ to E_T_. The reference samples (in air-dry condition) not exposed to high temperature were designated A_R_ to E_R_. In total, 50 samples (10 from each series) were tested. Figure 2 presents exemplary views of the tested 8 mm thick 20 × 100 mm samples.

**Figure 3:**
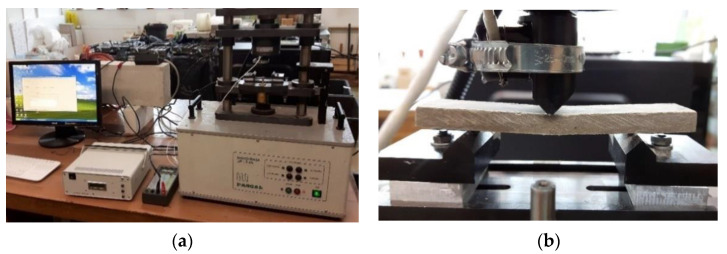
Test bench with equipment for acoustic emission measurements (**a**) and close-up of cement board sample during test (**b**).

In order to identify the effect of the high temperature on the structure of the FCB, the latter were investigated under three-point bending by means of acoustic emission. During the three-point bending, the trace of bending force *F*, strain *ε* and acoustic emission signals were registered. Figure 3 shows the three-point bending test bench with the acoustic emission measurement apparatus.

The analysis of the three-point test results covered the trace of flexural stress *σ*_m_, the bending strength (*MOR*), impact energy *W*_f_, the limit of proportionality (*LOP*) and strain *ε*. *MOR* was determined from the standard formula [46]:

(1) $$MOR=\frac{3Fl_{s}\;}{2b\;e^{2}},$$

where:

*F*—the ultimate force [N],
*l*_s_—the spacing of the supports [mm],
*b*—the width of the tested sample [mm],
*e*—the tested sample’s mean thickness measured in four places [mm].

The impact energy was determined using the following formula taken from [32]:

(2) $$W_{f}=\frac{1}{S}\int_{F0}^{F0.4max}F\;da,$$

where:

*S*—the sample’s cross-sectional area [m^2^],
*A*—the deformation during bending [m].

Because of the wide range of test results, only the results for two selected series of the tested fibre-cement boards are presented and analysed in the further part of this paper. Fibre-cement boards A and C were selected for further analysis. This choice was guided by the fact that they are panels for external applications as façade claddings (nowadays it is the most common way of using these panels). Moreover, in contrast to the B series boards, these boards are in the opinion of the authors more representative due to the differences in the obtained MOR flexural strength (about 30%). In turn, the D and E series panels are characterized by much worse strength parameters and are panels for internal applications. They also have a slightly different composition because they do not contain PVA fibres. This makes it difficult to compare them with the A–C series boards.

**Figure 4:**
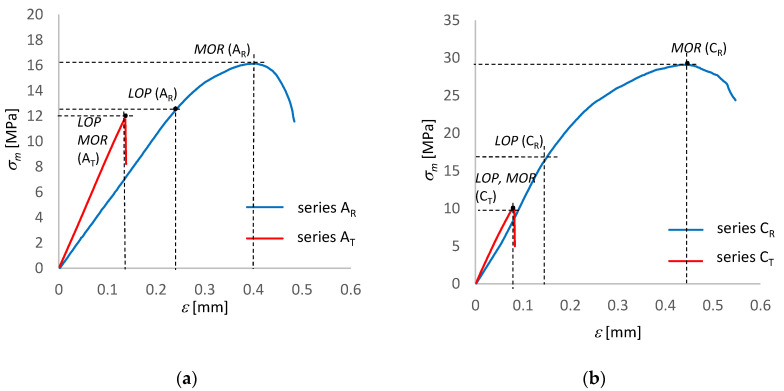
Bending *σ*–*ε* curves for fibre-cement boards of: (**a**) series A, (**b**) series C.

Figure 4 shows bending *σ*–*ε* curves for exemplary fibre-cement boards of series A and C. The *LOP* and *MOR* values are marked in the diagrams. Table 2 shows the results of the bending test to which the fibre-cement boards were subjected.

As it appears from Figure 4, the 3 h long influence of the temperature of 230 °C on *MOR* is clearly damaging for series A_T_ and C_T_. The tests showed that under the influence of the temperature *MOR* decreased by as much as 70%. An analysis of Table 2 shows that the fall in the value of impact energy *W*_f_ is very large. The value of this parameter is indicative of the changes which have taken place in the structure of the FCB under the influence of temperature, which manifest themselves by a fall in the energy needed to break the sample. It also appears from Table 2 that the value of Young’s modulus *E*_D_ increases by about 30% under the influence of a temperature of 230 °C lasting for 3 h. Moreover, it appears from the graphs presented in Figure 4 that the *σ*–*ε* dependence for the tested boards of series A_T_ and C_T_ also changes under the influence of high temperature. The influence of temperature manifests itself in not only in the decrease in *MOR*, but also in a change in the shape of the *σ–ε* curve. One can notice that *MOR* has become equal to *LOP*. In the case of the reference fibre-cement samples, the value of *MOR* is much higher. To sum up, it can be concluded from the decreased parameter values presented in Table 2 that damage occurred as a result of the exposure to a temperature of 230 °C for 3 h.

In order to better characterize the effect of high temperature on the structure of FCB, acoustic emission and artificial neural networks were employed.

## 4. Investigations Using Artificial Neural Networks and Acoustic Emission

As mentioned above, the next step in the study of the damaging effect of the high temperature on the structure of the FCB was an analysis of the AE signals recorded during three-point bending. Such AE descriptors as: events rate *N*_ev_, events sum ∑*N*_ev_, events energy *E*_ev_ and EA signal frequency distribution were used in the investigations. Table 3 shows exemplary values of events sum *∑N*_ev_ and events energy *E*_ev_ for series A_R_ and C_R_ in air-dry condition and for series A_T_ and E_T_ exposed to the temperature of 230 °C for 3 h. In addition, the average energy *E*_ev,avg_ of the events registered during the test was calculated. The analysis of the test results showed that EA signals were collected after the stress corresponding to *MOR* was exceeded.

The results contained in Table 3 indicate a marked fall in the number of events registered for the tested series of the boards exposed to the temperature of 230 °C for 3 h in comparison with the boards in air-dry condition. Moreover, it is apparent that the exposure to the temperature of 230 °C for 3 h results in a considerable increase in events energy *E*_ev_ during the bending test for all the tested series of FCB. The correlation between the events sum ∑*N*_ev_ descriptors and events energy *E*_ev_ indicates that the exposure to a temperature of 230 °C for 3 h results in an increase in energy *E*_ev_ of the registered events and a simultaneous reduction in their number. As a result of this exposure, the registered events come down to a single pulse induced by a brittle fracture. This is a high-energy event whose energy *E*_ev_ reaches 0.35 mJ.

**Figure 5:**
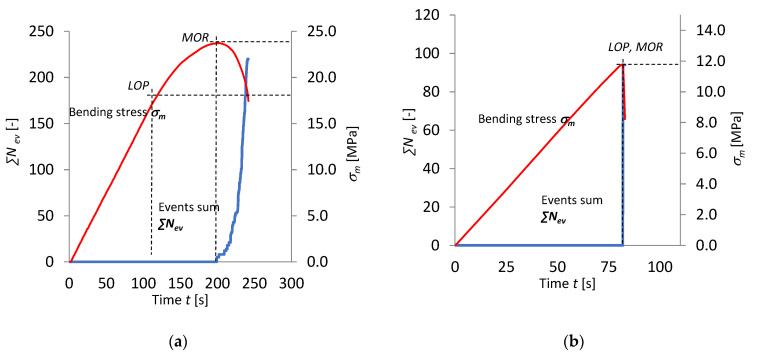
Bending stress *σ*_m_ and events sum ∑*N*_ev_ versus time *t* for boards of: (**a**) series A_R_ and (**b**) series A_T_.

**Figure 6:**
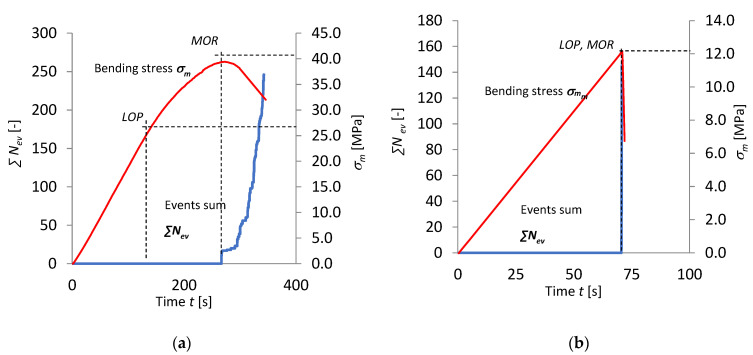
Bending stress *σ*_m_ and events sum ∑*N*_ev_ versus time *t* for boards of: (**a**) series C_R_ and (**b**) series C_T_.

In order to examine in more detail the course of the bending test and the effect of the damaging factor (the exposure to the high temperature of 230 °C for 3 h) on it, events sum ∑*N*_ev_ and bending stress *σ*_m_ versus time are presented graphically in Figure 5 and Figure 6.

A marked fall in the number of registered events and a change in the path of events sum ∑*N*_ev_ are visible in Figure 5 and Figure 6. Besides the fall in the number of events under the influence of the high temperature of 230 °C lasting for 3 h, it was noticed that all the registered events occurred within one time segment of 0.1 s. Hence, one can conclude that the events originate from a single fracture of the cement matrix. The damaging factor in the form of high temperature reduced the number of events, but resulted in an increase in their energy. The events registered for the cases in air-dry conditions, and which no longer registered in the boards after the latter had been baked at a temperature of 230 °C for 3 h, originate from the rupture of fibres damaged under the influence of the high temperature. The events registered for the boards of series A_T_ and C_T_ originate from the high-energy cracking of the cement matrix alone. In order to confirm the origin of the registered AE events, further investigations had to be made. A spectral analysis of the characteristics of the AE events spectra was carried out to identify the source of the registered AE events.

**Figure 7:**
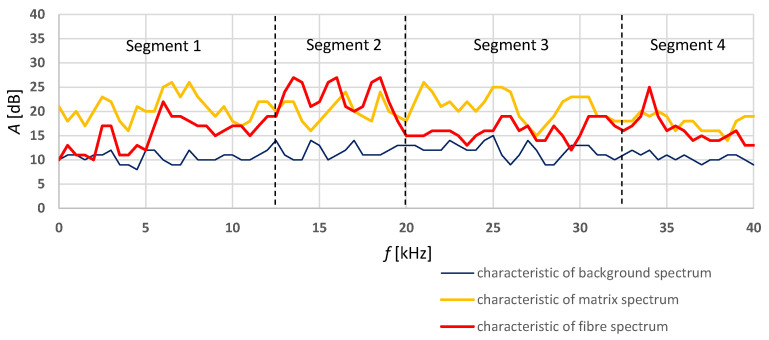
Background, fibre and cement matrix acoustic spectrum characteristics versus frequency.

Acoustic spectra models for the cracking of the cement matrix were selected on the basis of the analysis of the acoustic activity in the time-frequency domain during the bending of the boards in air-dry condition and the ones exposed to a temperature of 230 °C for 3 h. A model acoustic spectrum for the rupture of the fibres was selected from the spectra obtained for the boards in air-dry conditions. The model acoustic spectrum showed a repeatable similar pattern in the frequency range of 10–24 kHz, clearly distinct from that of the cement matrix spectrum. The characteristic of the acoustic spectrum of the background originating from the press was determined on the basis of the initial phase of bending by averaging the characteristics obtained for all the tested boards of series A and C. The selected spectral characteristics of a fibre rupture are understood as the signal accompanying the cracking of the cement matrix with fibres, while the model spectral characteristic of the matrix is understood as the signal accompanying the cracking of the cement matrix alone. The selected model acoustic spectrum characteristics were registered at every 0.5 kHz in 80 intervals. Figure 7 shows the record of the model spectrum characteristics of the signal accompanying the cracking of the cement matrix and the rupturing of the fibres, and of the background signal.

It appears from Figure 7 that the acoustic activity of the background is within 10–15 dB. The characteristic of the acoustic spectrum of the cement matrix reaches the acoustic activity of 25 dB in the ranges of 5–10 kHz (segment 1) and 20–32 kHz (segment 3). For the fibres, an activity level above 25 dB in the frequency ranges of 12–18 dB (segment 2) and 32–38 kHz (segment 4) was read. The model characteristic for the cement matrix, the fibres and the background were implemented in artificial neural networks and the training and testing of the latter began. A unidirectional multilayer structure with error backpropagation with momentum was selected for the ANNs [11,47,48]. Details concerning the selected ANNs and the training and testing procedures can be found in [49]. Having trained the ANN on the input data, the correctness of its mapping was checked on the training data and the testing data. Two pairs of input data, i.e., the data used for training the network and checking its ability to reproduce the model characteristics and the testing data used for checking the network’s ability to identify the model spectral characteristics originating from the fibres and the matrix respectively during the bending test were used for this purpose. Consequently, a record of the neural networks’ outputs of the recognized acoustic spectra corresponding to the fibre rupturing, matrix crack and the background respectively was obtained.

**Figure 8:**
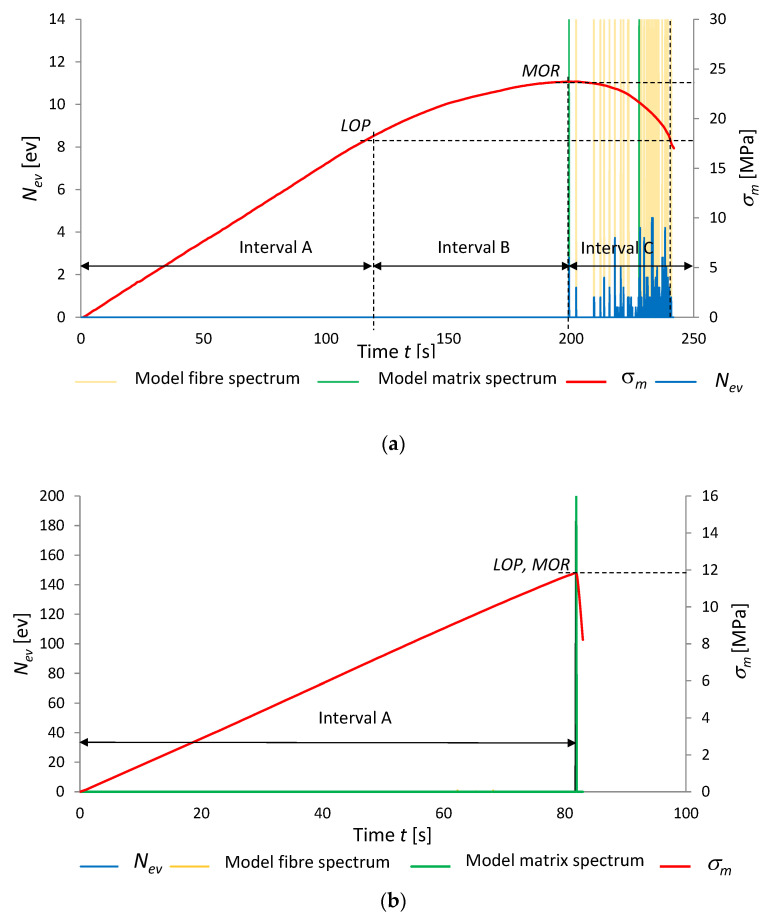
Events rate *N*_ev_ and bending stress *σ*_m_ versus time, under influence of fire, with marked identification of model spectral characteristics: (**a**) series A_R_, (**b**) series A_T_.

**Figure 9:**
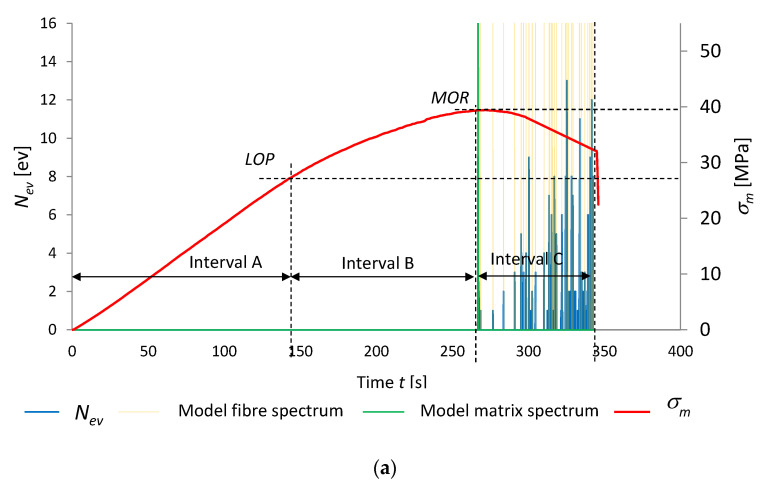

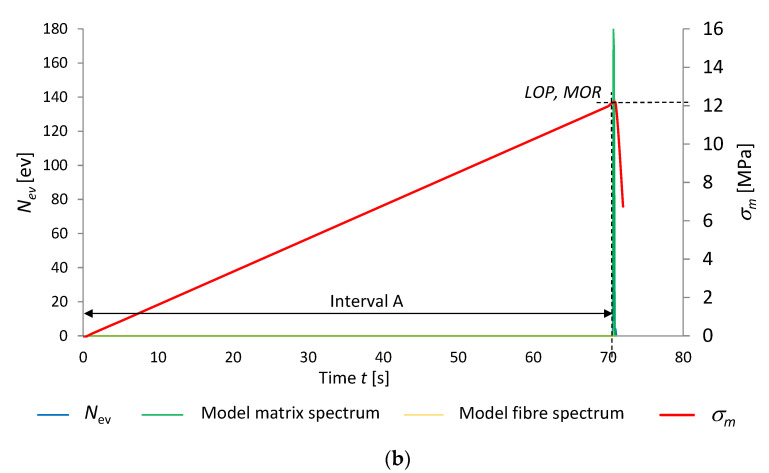
Events rate *N*_ev_, and bending stress *σ*_m_ versus time under influence of fire, with marked identification of model spectral characteristics: (**a**) series C_R_, (**b**) series C_T_.

Figure 8 and Figure 9 present the results of the recognition of the model acoustic spectra for the cement matrix and the fibres. They are marked on the record of events rate *N*_ev_ and bending stress *σ*_m_ as a function of time. The graphs are for the boards of series A_R_ and C_R_ in air-dry condition and series A_T_ and C_T_ exposed to the temperature of 230 °C for 3 h. For clarity, the recognized model acoustic spectra for the matrix are marked green and the ones for the fibres are marked orange.

Analysing Figure 8a, three intervals, A, B and C, could be distinguished. It appears from this figure that as late as in interval C (after *MOR* had been reached) AE events were registered. No AE events were registered in intervals A and B. The registered events successively dominate after the maximum tensile bending stress is exceeded. The recognized events in interval C originate from the rupturing of the fibres and the cracking of the cement matrix. An event originating from the cracking of the cement matrix initiates subsequent events originating from the rupturing of the fibres. The matrix fractures after the bending strength (*MOR*) is reached. It appears from Figure 8b that *MOR* has become equal to *LOP*. Interval B between *LOP* and *MOR* is missing, while interval C has been reduced to a single time segment of 0.1 s with registered AE events. The fact that *MOR* has become equal to *LOP* is evidence of changes in the board’s structure which occurred under the influence of high temperature. Whereas the reduction of interval C to a single segment is indicative of a single signal of the brittle fracture of the fibre-cement board and of the absence of fibres in its structure, which could carry tensile stresses. The acoustic spectrum characteristic of the events registered in interval C was recognized as the model spectrum of the cement matrix.

An exemplary breakdown of the events recognized as accompanying the rupturing of fibres and the cracking of the cement matrix for series A_R_ and A_T_ is presented in Table 4.

The results presented in Table 4 indicate that no events originating from the rupturing of fibres were registered in the case of the boards exposed to the high temperature, which is clear evidence of the total failure of the fibres. The events which were not recognized during the testing of the fibre-cement boards in air-dry condition had too low event energy *E*_ev_ or their acoustic spectrum characteristics were atypical and at the similarity classifier of 0.9 were not assigned to the selected characteristics. The increase in the number of events ascribed to the cracking of the cement matrix in the boards exposed to the high temperature is due to the extreme event energy *E*_ev_ accompanying the brittle fracture.

Figure 9a shows that the course of bending and that of the AE signals registered for the boards of series C_R_ are similar to those for series A_R_. Three intervals can be distinguished in the bending stress diagram. Numerous AE signals predominate in the last interval. The boards of series C_R_ have a large number of fibres in their structure. It appears from the figure that there is no interval B, while interval C containing AE signals has been reduced to a single time segment of 0.1 s, in which the recognized brittle fracture pattern was registered. The course of the bending test and that of AE descriptors are similar to those for the boards of series A_T_. A comparison of Figure 9a and Figure 9b clearly shows that exposure to a high temperature of 230 °C for 3 h is a damaging factor causing the degradation of the fibres contained in the board. This fact is very well reflected by the results presented in Table 5 in which the events recognized as accompanying the rupturing of fibres and the cracking of the cement matrix are collated for the fibre-cement boards of series C_R_ and C_T_.

Table 5 shows an exemplary comparison of the events recognized as accompanying the rupturing of fibres and the cracking of the cement matrix for series C_R_ and C_T_.

It appears from Table 5 that the fibre-cement boards of series C_R_ are characterized by a large number of events originating from the rupturing of fibres. These boards show the highest *MOR* from the tested series.

To sum up, it should be noted that the boards of series A_R_ and C_R_ in air-dry conditions are characterized by a similar bending stress diagram in which three intervals can be distinguished. The fact that high-energy AE events are registered signals possible differences between the reference boards and the ones subjected to damaging factors. By identifying the acoustic spectrum characteristics through reading and assigning them to the cement matrix or the fibres, it became possible to accurately identify the damage which takes place in the boards under the influence of temperature.

No acoustic spectrum characteristics corresponding to fibres were recognized in the boards of series A_T_ and C_T_ exposed to the temperature of 230 °C for 3 h. This confirms the fact that the fibres undergo degradation at the high temperature of 230 °C. They fracture brittlely under the influence of the high temperature. Analysing AE signals and identifying the acoustic spectrum characteristics, one can assess damage to the structure of the boards, especially damage to the fibres contained in fibre-cement boards. In order to verify the above conclusions, the authors decided to carry out additional optical investigations using a scanning electron microscope (*SEM*) to compare the structure of the tested boards.

## 5. SEM Examinations

The optical investigations by means of a scanning electron microscope with EDS analyser were carried out in collaboration with the Faculty of Civil Engineering and Architecture at Kielce University of Technology.

**Figure 10:**
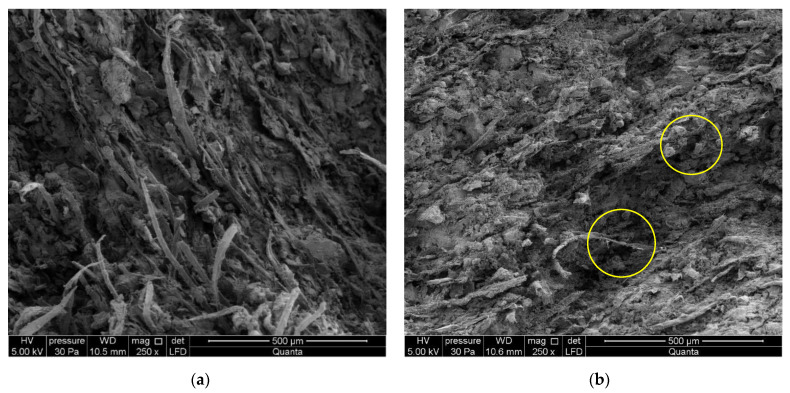

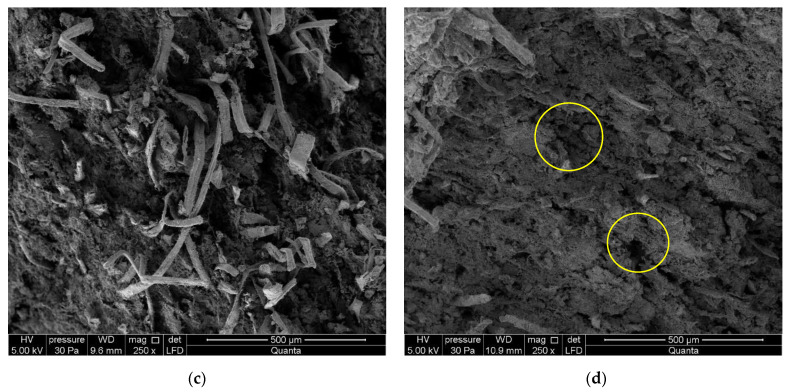
SEM images of boards of series: (**a**) A_R_, (**b**) A_T_, (**c**) C_R_, (**d**) C_T_.

The boards of series A_R_ and C_R_ in air-dry condition and the boards of series A_T_ and C_T_ exposed to a temperature of 230 °C for 3 h were subjected to SEM examinations. Figure 10 shows example images for series A and C.

On the basis of the images shown in Figure 10 one can describe the macrostructure of the tested fibre-cement boards as compact. The SEM examinations revealed the structure to be fine pore, with the pores up to 50 µm in size. Cavities and grooves left by the pulled-out fibres were visible in the fracture area. Cellulose fibres and PVA fibres are clearly visible in the images, as shown in Figure 10a,c. Various forms of calcium silicate hydrates of the C-S-H type occur, with an amorphous phase and a phase made up of strongly adherent particles predominating. An EDS analysis of the cement matrix’s chemical composition showed elements consistent with the composition of the cement. The fibres are covered with a thin layer of cement paste and hydration products. The fact that there are few places with a space between the fibres and the matrix is indicative of a strong bond between them. The examination of the fibre-cement boards of series A_T_ and C_T_ exposed to a temperature of 230 °C for 3 h revealed a distinct change in the colour of the samples at the macroscopic level. Most of the fibres in the boards were found to be burnt out or fused into the cement matrix, leaving cavities and grooves, as shown in Figure 10b,d (some such places are marked with a yellow circle). The structure of the few remaining fibres was heavily degraded. Examinations of the cement grains in other fractured surfaces also revealed that their structure was damaged by the high temperature. The structure of the cement matrix was found to be highly granular with numerous delaminations. More numerous cavities and grooves left after the pulled-out fibres and grooves left after the pulled-out cement grains were observed.

Summing up the examinations carried out using the optical method, we note that this method makes it possible to very precisely describe the inner structure of fibre-cement boards. In the case of the boards exposed to high temperature, most of the fibres were found to be burnt out or fused into the matrix, leaving cavities and grooves. However, significantly, in the case of the reference fibre-cement boards there are many more ruptured fibres whose ends are well “anchored” in the cement matrix. This clearly means that exposure to a temperature of 230 °C for 3 h has a damaging effect on the structure of fibre-cement boards.

## 6. Conclusions

Exposure to high temperature is by nature damaging to most building products. The tests have shown that the exposure of a fibre-cement board to a temperature of 230 °C for 3 h is critical as it results in the total destruction of the board. The following conclusions emerge from the investigations:

1. The influence of the temperature of 230 °C for 3 h on bending strength (*MOR*) is clearly damaging for series A_T_ and C_T_. Under the influence of the temperature *MOR* decreased by as much as 70%. In the case of these boards, impact energy *W*_f_ also decreased considerably, while Young’s modulus *E*_D_ increased by 30%.
2. The *σ*–*ε*, curves indicated that the influence of the temperature results not only in a decrease in *MOR*, but also in a change in the shape of the *σ*–*ε* curve. Moreover, *MOR* becomes equal to *LOP*. In the case of the reference fibre-cement boards, the value of *MOR* is much higher.
3. The acoustic emission investigations during the three-point bending test showed a marked fall in the number of AE events registered for the tested series of boards under the influence of the high temperature in comparison with the reference boards. Moreover, it was noticed that the exposure to the temperature of 230 °C for 3 h results in a considerable increase in events energy *E*_ev_ during the bending test for the tested series of fibre-cement boards, whereby the registered events, by and large, come down to a single pulse induced by a brittle fracture.
4. The results yielded by the identification of the model spectral characteristics by means of ANNs indicate that the boards of series A_R_ and C_R_ in air-dry condition are characterized by a similar bending stress diagram in which three intervals can be distinguished. The acoustic spectrum characteristics corresponding to the fibres and the cement matrix respectively were identified. However, no acoustic spectrum characteristics corresponding to the fibres were identified in the boards of series A_T_ and C_T_ exposed to the temperature of 230 °C for 3 h. This confirms the fact that at high temperatures, the fibres undergo degradation. Under the influence of high temperature, the boards fracture brittlely.
5. The optical examinations under a SEM with an EDS analyser revealed that in the boards exposed to the high temperature, most of the fibres had been burnt out or fused into the cement matrix, leaving cavities and grooves. In the case of the reference boards, a large number of fibres whose ends were well “anchored” in the cement matrix were ruptured on the fractured surfaces.

Summing up the above conclusions drawn from the experiments carried out as part of this research, one can definitely state that exposure to a temperature of 230 °C for 3 h has a damaging effect on the structure of fibre-cement boards. In the authors’ opinion, the findings are important for building practice as, so far, there has been little information about the behaviour of rainscreen cladding made of fibre-cement boards exposed to high temperatures. It is also worth noting that exterior wall cladding is exposed to high temperature not only in the case of a fire, but also through insolation. In the authors’ opinion, it would be worth to carry out ageing tests of fibre-cement boards subjected to UV radiation. This will certainly be the subject of further research.

Finally, it is worth noting that the research carried out in this paper clearly confirmed the usefulness of the acoustic emission method and artificial neural networks for the assessment of the impact of the destructive effect of high temperature on the structure of fiber-cement boards. According to the authors, the use of the method of acoustic emission and artificial neural networks to assess the destructive effect of high temperature on the structure of fiber-cement boards is a unique measuring technique that can play a large role in construction practice today or in the near future. In addition, it is worth adding that the new approach to measuring cracks, deformations or acoustic measurements in materials at elevated temperatures certainly has great application in other materials. This approach can be used to monitor the development of microcracks or scratches in materials in order to control their strength and thus create new safer composites with a view to fire safety.

## Figures and Tables

**Table 1:** Tested fibre-cement boards of series A to E and their basic specifications.

Series Designation	Board Thickness *e* [mm]	Board Colour	Application	Board Bulk Density *ρ* [g/cm^3^]	Bending Strength *MOR* [MPa]
A	8.0	natural	exterior	1.60	23.1
B	8.0	full body coloured	exterior	1.60	30.4
C	8.0	full body coloured	exterior	1.65	32.3
D	8.0	natural	interior	1.70	20.5
E	8.0	natural	interior	1.20	12.7

**Table 2:** Tested FCB of series A and C and their basic specifications.

Board Series Designation	Ultimate Force *F* [N]	LOP [MPa]	Young’s Modulus *E_D_* [GPa]	Impact Energy *W_f_* [J/m^2^]	*MOR* [MPa]
Series A_R_	252.26	15.5	6.0	391.82	20.73
				(3.45%) *	(2.39%) *
Series A_T_	82.36	6.77	7.5	42.99	6.77
				(3.87%) *	(3.44%) *
Series C_R_	475.18	24.20	8.1	1352.60	35.60
				(1.94%) *	(3.98%) *
Series C_T_	115.28	8.64	10.4	45.44	8.64
				(3.51%) *	(3.14%) *

* Note: The coefficient of variation is in brackets.

**Table 3:** Summary of representative values of events sum ∑*N*_ev_, events maximum energy *E*_ev_ and events average energy *E*_ev,avg_ for boards of series A_R_ and C_R_ in dry-air condition and series A_T_ and C_T_ exposed to temperature of 230 °C for 3 h.

Board Series Designation	Events Sum ∑*N*_ev_ [ev]	Events Energy *E*_ev_ [nJ]	Events Average Energy *E*_ev,avg_ [nJ]
Series A_R_	223.0	4214.0	18.89
Series A_T_	111.0	31,6765.0	1853.74
Series C_R_	496.0	18,338.0	36.97
Series C_T_	142.0	348,255.0	2452.50

**Table 4:** Events recognized as accompanying rupturing of fibres and cracking of cement matrix for boards of series A_R_ and A_T_.

Series	Events Sum ∑*N*_ev_	Total Number of Recognized Events ∑*N*_ev,r_	Total Number of Events Ascribed to Fibre Rupturing ∑*N*_ev,f_	Total Number of Events Ascribed to Matrix Cracking ∑*N*_ev,m_
Series A_R_	223	203	195	8
Series A_T_	94	94	0	94

**Table 5:** Events recognized as accompanying the rupturing of fibres and cracking of the cement matrix for boards of series C_R_ and C_T_.

Series	Events Sum ∑*N*_ev_	Total Number of Recognized Events ∑*N*_ev,r_	Total Number of Events Ascribed to Fibre Rupturing ∑*N*_ev,f_	Total Number of Events Ascribed to Matrix Cracking ∑*N*_ev,m_
Series C_R_	496	483	445	38
Series C_T_	142	142	0	142

## Data Availability

Not applicable.

## References

[1] Schabowicz K., Gorzelańczyk T., Ranachowski Z., Schabowicz K. (2018). Fabrication of fibre cement boards. The Fabrication, Testing and Application of Fibre Cement Boards.

[2] Bentchikou M., Guidoum A., Scrivener K., Silhadi K., Hanini S. (2012). Effect of recycled cellulose fibres on the properties of lightweight cement composite matrix. Constr. Build. Mater..

[3] Savastano H., Warden P.G., Coutts R.S.P. (2005). Microstructure and mechanical properties of waste fibre–cement composites. Cem. Concr. Compos..

[4] Coutts R.S.P. (2005). A Review of Australian Research into Natural Fibre Cement Composites. Cem. Concr. Compos..

[5] Schabowicz K., Szymków M. (2016). Ventilated facades made of fibre-cement boards. Mater. Bud..

[6] Ardanuy M., Claramunt J., Toledo Filho R.D. (2015). Cellulosic Fibre Reinforced Cement-Based Composites: A Review of Recent Research. Constr. Build. Mater..

[7] Drelich R., Gorzelanczyk T., Pakuła M., Schabowicz K. (2015). Automated control of cellulose fibre cement boards with a non-contact ultrasound scanner. Autom. Constr..

[8] Dębowski T., Lewandowski M., Mackiewicz S., Ranachowski Z., Schabowicz K. (2016). Ultrasonic tests of fibre-cement boards. Przegląd Spaw..

[9] Schabowicz K., Jóźwiak-Niedźwiedzka D., Ranachowski Z., Kudela S., Dvorak T. (2018). Microstructural characterization of cellulose fibres in reinforced cement boards. Arch. Civ. Mech. Eng..

[10] Chady T., Schabowicz K., Szymków M. (2018). Automated multisource electromagnetic inspection of fibre-cement boards. Autom. Constr..

[11] Schabowicz K., Gorzelańczyk T., Szymków M. (2019). Identification of the degree of fibre-cement boards degradation under the influence of high temperature. Autom. Constr..

[12] Schabowicz K., Gorzelańczyk T., Szymków M. (2019). Identification of the degree of degradation of fibre-cement boards exposed to fire by means of the acoustic emission method and artificial neural networks. Materials.

[13] Zhang C., Chao L., Zhang Z., Zhang L., Li Q., Fan H., Zhang S., Liu Q., Qiao Y., Tian Y. (2021). Pyrolysis of cellulose: Evolution of functionalities and structure of bio-char versus temperature. Renew. Sustain. Energy Rev..

[14] Leflik M. (2013). Some aspects of application of artificial neural network for numerical modeling in civil engineering. Bull. Pol. Acad. Sci. Tech. Sci..

[15] Gorzelańczyk T., Schabowicz K., Szymków M. (2016). Non-destructive testing of fibre-cement boards, using acoustic emission. Przegląd Spaw..

[16] Adamczak-Bugno A., Gorzelańczyk T., Krampikowska A., Szymków M. (2017). Non-destructive testing of the structure of fibre-cement materials by means of a scanning electron microscope. Bad. Nieniszcz. I Diagn..

[17] Claramunt J., Ardanuy M., García-Hortal J.A. (2010). Effect of drying and rewetting cycles on the structure and physicochemical characteristics of softwood fibres for reinforcement of cementitious composites. Carbohydr. Polym..

[18] Mohr B.J., Nanko H., Kurtis K.E. (2005). Durability of kraft pulp fibre-cement composites to wet/dry cycling. Cem. Concr. Compos..

[19] Pizzol V.D., Mendes L.M., Savastano H., Frías M., Davila F.J., Cincotto M.A., John V.M., Tonoli G.H.D. (2014). Mineralogical and microstructural changes promoted by accelerated carbonation and ageing cycles of hybrid fibre–cement composites. Constr. Build. Mater..

[20] Gorzelańczyk T., Pachnicz M., Różański A., Schabowicz K. (2019). Multi-Scale Structural Assessment of Cellulose Fibres Cement Boards Subjected to High Temperature Treatment. Materials.

[21] Gorzelańczyk T., Pachnicz M., Różański A., Schabowicz K. (2020). Identification of microstructural anisotropy of cellulose cement boards by means of nanoindentation. Constr. Build. Mater..

[22] Li Z., Zhou X., Bin S. (2004). Fibre-Cement extrudates with perlite subjected to high temperatures. J. Mater. Civ. Eng..

[23] Adamczak-Bugno A., Krampikowska A., Świt G. (2021). Analysis of the Frequency of Acoustic Emission Events in Terms of the Assessment of the Reduction of Mechanical Parameters of Cellulose–Cement Composites. Materials.

[24] Schabowicz K., Gorzelańczyk T. (2016). A non-destructive methodology for the testing of fibre cement boards by means of a non-contact ultrasound scanner. Constr. Build. Mater..

[25] Stark W., Karbhari V.M. (2013). Non-destructive evaluation (NDE) of composites: Using ultrasound to monitor the curing of composites. Non-destructive Evaluation (NDE) of Polymer Matrix Composites. Techniques and Applications.

[26] Berkowski P., Dmochowski G., Grosel J., Schabowicz K., Wójcicki Z. (2013). Analysis of failure conditions for a dynamically loaded composite floor system of an industrial building. J. Civ. Eng. Manag..

[27] Hoła J., Schabowicz K. (2010). State-of-the-art non-destructive methods for diagnostic testing of building structures–anticipated development trends. Arch. Civ. Mech. Eng..

[28] Davis A., Hertlein B., Lim K., Michols K. (1996). Impact-Echo and Impulse Response Stress WAVE methods: Advantages and limitations for the Evaluation of Highway Pavement Concrete Overlays. Proceedings of the Conference on Nondestructive Evaluation of Bridges and Highways.

[29] Chady T., Schabowicz K. (2016). Non-destructive testing of fibre-cement boards, using terahertz spectroscopy in time domain. Bad. Nieniszcz. I Diagn..

[30] Schabowicz K., Ranachowski Z., Jóźwiak-Niedźwiedzka D., Radzik Ł., Kudela S., Dvorak T. (2016). Application of X-ray microtomography to quality assessment of fibre cement boards. Constr. Build. Mater..

[31] Ranachowski Z., Ranachowski P., Dębowski T., Gorzelańczyk T., Schabowicz K. (2019). Investigation of structural degradation of fiber cement boards due to thermal impact. Materials.

[32] Ranachowski Z., Schabowicz K. (2017). The contribution of fibre reinforcement system to the overall toughness of cellulose fibre concrete panels. Constr. Build. Mater..

[33] Ranachowski Z. (1996). The application of neural networks to classify the acoustic emission waveforms emitted by the concrete under thermal stress. Arch. Acoust..

[34] Ranachowski Z., Jóźwiak-Niedźwiedzka D., Brandt A.M., Dębowski T. (2012). Application of acoustic emission method to determine critical stress in fibre reinforced mortar beams. Arch. Acoust..

[35] Melichar T., Bydzovsky J., Dvorak R., Topolar L., Keprdova S. (2021). The Behavior of Cement-Bonded Particleboard with Modified Composition under Static Load Stress. Materials.

[36] Yuki H., Homma K. (1996). Estimation of acoustic emission source waveform of fracture using a neural network. NDT E Int..

[37] Schabowicz K. (2005). Neural networks in the NDT identification of the strength of concrete. Arch. Civ. Eng..

[38] Asteris P.G., Kolovos K.G. (2019). Self-compacting concrete strength prediction using surrogate models. Neural Comput. Appl..

[39] Lee S.C. (2003). Prediction of concrete strength using artificial neural networks. Eng. Struct..

[40] Łazarska M., Woźniak T., Ranachowski Z., Trafarski A., Domek G. (2017). Analysis of acoustic emission signals at austempering of steels using neural networks. Met. Mater. Int..

[41] Woźniak T.Z., Ranachowski Z., Ranachowski P., Ozgowicz W., Trafarski A. (2014). The application of neural networks for studying phase transformation by the method of acoustic emission in bearing steel. Arch. Metall. Mater..

[42] Rucka M., Wilde K. (2013). Experimental study on ultrasonic monitoring of splitting failure in reinforced concrete. J. Nondestruct. Eval..

[43] Rucka M., Wilde K. (2015). Ultrasound monitoring for evaluation of damage in reinforced concrete. Bull. Pol. Acad. Sci. Tech. Sci..

[44] Zielińska M., Rucka M. (2018). Non-Destructive Assessment of Masonry Pillars using Ultrasonic Tomography. Materials.

[45] Wojtczak E., Rucka M. (2019). Wave Frequency Effects on Damage Imaging in Adhesive Joints Using Lamb Waves and RMS. Materials.

[46] (2018). EN 12467—Cellulose Fibre Cement Flat Sheets. Product Specification and Test Methods. https://standards.cen.eu/dyn/www/f?p=204:110:0::::FSP_PRJECT,FSP_ORG_ID:66671,6110&cs=1151E39EDCD9EF75E3C2D401EB5818ACD.

[47] Osowski S. (2000). Neural Networks for Information Processing.

[48] Estêvão J.M.C. (2018). Feasibility of using neural networks to obtain simplified capacity curves for seismic assessment. Buildings.

[49] Gorzelańczyk T., Schabowicz K. (2019). Effect of Freeze–Thaw Cycling on the Failure of Fibre-Cement Boards, Assessed Using Acoustic Emission Method and Artificial Neural Network. Materials.

